# High-Temperature Deformation Behaviors of Gradient-Structured Mg-Gd-Y-Zr Alloys at High Strain Rates

**DOI:** 10.3390/ma18174085

**Published:** 2025-08-31

**Authors:** Jialiao Zhou, Minghui Wu, Wenxuan Zhang, Jiangli Ning

**Affiliations:** 1 Innovation Center for New Materials and Processing Technology, Ningbo Institute of Dalian University of Technology, Ningbo 315000, China; 2 Key Laboratory of the Ministry of Education for Modern Metallurgy Technology, North China University of Science and Technology, Tangshan 063210, China; 3 State Key Laboratory of Powder Metallurgy, Central South University, Changsha 410083, China

**Keywords:** Mg-Gd-Y-Zr alloy, gradient structure, high-temperature deformation behavior, dynamic recrystallization, grain boundary slip

## Abstract

The deformation behaviors of a gradient-structured (GS) Mg-Gd-Y-Zr alloy, prepared via surface mechanical attrition treatment (SMAT), were systematically investigated in comparison with those of a uniform coarse-grained (CG) counterpart by high-temperature tensile tests at high strain rates (≤400 °C and ≥0.01 s^−1^). The results indicated that the uniform CG samples exhibited high flow stresses and low elongations (43.9% at 400 °C and 0.01 s^−1^). Their fraction of dynamic recrystallization (DRX) during the hot deformation was very low, and the dislocations accumulated inside the deformed grains formed high residual stresses. Moreover, the solely operated prismatic <a> slips in the coarse grains implied insufficient deformation coordination. These resulted in their low deformability. By contrast, the GS samples formed by SMAT exhibited more stable flow behaviors, showing lower flow stresses and higher elongations (71.9% at 400 °C and 0.01 s^−1^). The high dislocation density in the severely deformed (SD) layer provided sufficient driving force for DRX, promoting remarkable softening effect during the hot deformation. The grain boundary slip mechanism facilitated by DRX in the SD layer played a significant role in the hot deformation, enhancing the overall plasticity of the GS samples, although the deformed coarse-grained (DCG) layer deformed in a manner resembling that of the CG samples.

## 1. Introduction

As a class of crucial lightweight metallic materials, magnesium alloys have attracted significant attention and found widespread applications in various industrial sectors. The unique physical and mechanical properties, particularly their low density and high specific strength, make them an ideal choice for lightweight design [1,2,3]. However, due to the hexagonal close-packed (HCP) crystal structure with a c/a ratio of 1.624, the deformation of magnesium alloys at room temperature primarily relies on basal slip. The limited number of slip systems leads to poor formability at room temperature [4]. In order to activate non-basal slip systems and accommodate plastic strain, plastic processing of magnesium alloys is feasibly performed at elevated temperatures [5].

Magnesium alloys can be used to prepare complex-shaped components through superplastic forming, but the high process cost limits the wide application of this technique. Before superplastic forming, pre-treatment is generally conducted on magnesium alloys with severe deformation to obtain a uniform fine-grained microstructure with an average grain size of less than 10–15 μm [6]. Additionally, the superplastic forming normally requires deformation conditions of high temperatures (>400 °C) and low strain rates (≤10^−3^ s^−1^) [7,8], which will inevitably cause high energy consumption. Wu et al. [9] demonstrated that a superplastic elongation of 726% at a strain rate of 0.01 s^−1^ was achieved in an Mg-6.5Y-1.2Er-1.6Zn-0.5Ag alloy after ECAP processing, which introduced 18R-LPSO and γ nano-precipitates and refined the grain size to approximately 2.9 µm. In another study on a WEZ612-1Ag alloy containing 1 wt% Ag [10], they attributed an increased elongation of 495% at the same strain rate to a non-basal texture and a high density of basal γ phase. However, the enhancement still relied on the presence of a submicron grain structure. Vávra et al. [11] obtained an ultra-high elongation of ~1230% at 0.01 s^−1^ in a WE43 (Mg-4Y-3RE) alloy after refining its grain size to ~340 nm via eight ECAP passes. Their work emphasized that grain size, in conjunction with thermally stable Mg_5_RE particles, determines the superplastic limit at high strain rates. Conversely, Zhan et al. [12] reported a much lower elongation of 442.49% at 0.01 s^−1^ and 450 °C for a conventionally hot-extruded Mg-2.5Nd-0.5Zn-0.5Zr alloy with an initial grain size of 2.78 µm. It confirms that conventionally deformed fine grains are insufficient to meet the requirements for superplasticity at high strain rates. Therefore, it is of significant engineering importance to develop a high-efficiency, low-cost (low temperature and high strain rate) plastic forming path for magnesium alloys that does not rely on uniform fine-grained microstructures.

In recent years, Surface Mechanical Attrition Treatment (SMAT) has been widely reported as an effective method for reducing grain size and obtaining gradient structures [13,14]. Xia et al. [15] showed that applying SMAT to Mg-10Gd-3Y-0.4Zr alloy enhanced precipitation hardening, substantially improved the hardness and strength through the introduction of a high dislocation density. Sun et al. [16] successfully prepared a gradient nanostructured surface layer on Mg-Y-Nd-Gd-Zr alloy using SMAT. This structure achieved a combination of high strength and good ductility through the synergistic effects of grain refinement, dislocation hardening, and supersaturated solid solution hardening. Although the preparation of gradient structures (GS) via SMAT has been extensively studied to improve the room-temperature mechanical properties of magnesium alloys, there are few reports concerning the deformation behavior and microstructure evolution of GS magnesium alloys during high-temperature tensile tests. Also the high-temperature deformation mechanisms of GS magnesium alloys are currently unclear.

In this study, two types of Mg-8.5Gd-2.5Y-0.5Zr alloys with uniform coarse grain (CG) and gradient structures were fabricated by Extrusion + Solution treatment (ES) and Extrusion + Solution treatment + SMAT (ESS) procedures, respectively. Subsequently, high-temperature tensile tests under the conditions of ≤400 °C and ≥0.01 s^−1^ were carried out to study the deformation behaviors of these alloys at relatively low temperatures and high strain rates. Electron backscatter diffraction (EBSD) analyses were performed to elucidate the hot deformation mechanisms of the ES and ESS samples and reveal the effects of temperature and strain rate on the microstructure evolution of the alloys.

## 2. Materials and Experimental Procedures

The Mg-8.5Gd-2.5Y-0.5Zr (wt%) alloy was manufactured by a resistance furnace using a semi-continuous casting method in a mixed protective atmosphere of CO_2_ and SF_6_ (volume ratio 99:1). The primary raw materials were high-purity Mg (99.96%), Mg-Gd, and Mg-Y intermediate alloys. When the melting was finished, the melt was immediately poured into a steel mold preheated to 200 °C to obtain an Mg-Gd-Y-Zr ingot with a diameter of 125 mm. The chemical composition was measured using an Inductively Coupled Plasma Atomic Emission Spectrometer (ICP-AES), as shown in Table 1. The Mg-Gd-Y-Zr ingot was subjected to homogenization treatment at 525 °C for 12 h. Subsequently, hot extrusion was conducted at 450 °C with an extrusion ratio of 16:1 and an extrusion speed of 0.3 mm·s^−1^, then cooled rapidly with strong air cooling. The ultimate diameter of the extruded rod was 32 mm.

Once the hot extrusion was finished, two sets of rectangular samples with a thickness of 5 ± 0.5 cm are cut from the extruded rods. And both sets of samples underwent solution treatment at 500 °C for 4 h. After that, Surface Mechanical Attrition Treatment (SMAT) was applied to one set of samples at a frequency of 50 Hz using a SPEX 8000 M Mixer/Mill. During the process, the disk-shaped samples were subjected to impacts from 40 stainless steel balls, each 6 mm in diameter, for 60 min within an argon-filled chamber to prevent oxidation. The processing and heat treatment operation steps of the samples are shown in Table 2, and in terms of the procedures, the two sets of samples are designated as ES and ESS, respectively.

The tensile test samples were prepared using wire electrical discharge machining with the gauge dimensions of 10 mm × 5 mm × 0.6 mm. Prior to the tensile tests, the samples were subjected to mechanical polishing, and then high-temperature tensile tests were performed on a universal testing machine (AG-X plus, 100 kN), with deformation temperatures of 350 °C and 400 °C, and strain rates of 0.01 s^−1^, 0.1 s^−1^, 0.5 s^−1^, and 1 s^−1^, respectively. All specimens were machined with the tensile direction parallel to the extrusion direction (ED). This is because a strong basal texture formed during the extrusion process, the tensile direction of the specimens is generally perpendicular to the (0001) basal plane texture. This alignment ensures consistency in evaluating the anisotropic deformation behavior of the textured material. Note that at least two tensile tests were conducted for each deformation parameter to minimize experimental errors. Besides, the necking occurring during the tensile process of all the samples took place within the gauge length. Regarding the narrowing of the cross-section, it is positively correlated with the elongation, meaning that the higher the elongation, the more significant the cross-sectional narrowing. The samples for electron backscatter diffraction (EBSD) analysis were mechanically ground and electropolished in AC2 electrolyte under a voltage of 20 V for 90 s. The EBSD data were collected at 20 kV with 1 μm scanning step size, and then analyzed with Channel 5 software.

## 3. Results

### 3.1. Hot Deformation Behaviors

**Figure 1:**
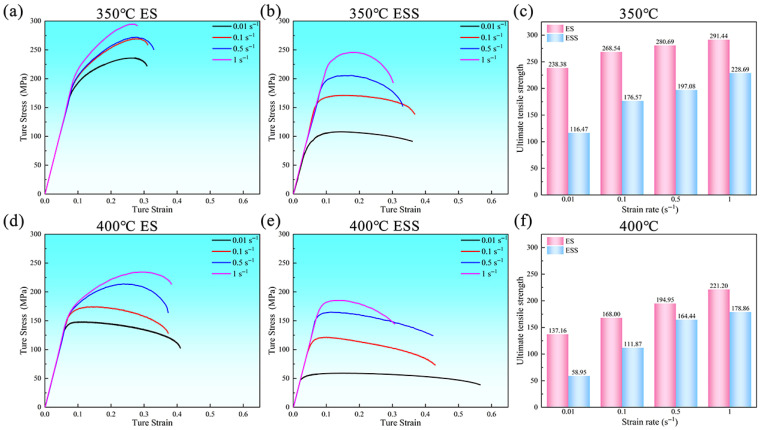
True stress-true strain curves: (**a**,**d**) True Stress-True Strain Curves of ES at 350 °C and 400 °C under Different Strain Rates; (**b**,**e**) True Stress-True Strain Curves of ESS at 350 °C and 400 °C under Different Strain Rates; (**c**,**f**) Comparison of the ultimate tensile strength of ES and ESS samples at 350 °C and 400 °C.

Figure 1 shows the true stress-true strain curves and variation in peak stress for ES and ESS samples under various deformation conditions (Figure 1a,b,d,e). The true stress values in this study were calculated by converting the engineering stress-strain curves. The conversion formulas used are as follows:

(1) $$\sigma_{t}=\sigma_{e}\times (1+\varepsilon_{e})$$

(2) $$\varepsilon_{t}=\ln(1+\varepsilon_{e})$$

where engineering stress (*σ_e_*) is obtained by dividing the applied load by the original cross-sectional area of the specimen, and engineering strain (*ε_e_*) is the ratio of the change in length to the original length of the specimen gauge during the tensile test. It can be observed that the true stress decreased with increasing temperature and decreasing strain rate. Particularly at 350 °C (Figure 1a), the true stress of ES samples showed a rapid increase as the true strain increased until reaching the peak stresses, implying remarkable strain hardening. After the peak stresses, the true stress decreased rapidly, and the samples fractured quickly. By contrast, the true stress-strain curves for the ESS samples exhibited gradual decline after rapidly reaching the peak stresses (Figure 1b), suggesting more notable softening effect overall during the deformation process [17]. Unlike the case at 350 °C, the ES specimen at 400 °C only exhibited a distinct work hardening stage at strain rates of 0.5 s^−1^ and 1 s^−1^, while this feature was less obvious at lower strain rates (Figure 1d). Furthermore, the true stress of the ESS specimen at this temperature increased more gradually with true strain during the elastic stage (Figure 1e). Notably, the true stress-strain curves under different strain rates rapidly reached the peak stress immediately after the elastic stage, followed by a slow and gradual decline in true stress with further increase in true strain until fracture. It is worth emphasizing that the peak stress of the ES specimen was consistently higher than that of the ESS specimen under all deformation conditions, indicating that the ES specimen possessed stronger work hardening capacity during deformation (Figure 1c,f).

**Figure 2:**
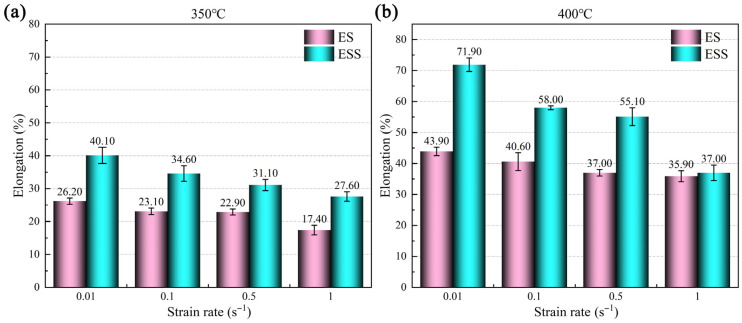
Comparison of elongation at break of ES and ESS alloys at different temperatures: (**a**) 350 °C; (**b**) 400 °C.

Figure 2 shows the values of fracture elongation of the ES and ESS samples under different deformation conditions. It can be seen that the fracture elongation of both types of samples increased as temperature rose, while it decreased as strain rate increased. When the temperature rose from 350 to 400 °C, the fracture elongation showed the most remarkable increase. In addition, under the same tensile parameters, the fracture elongation values of the ESS sample were higher than those of the ES samples.

Generally, the strain rate sensitivity (*m*) reflects the sensitivity of the flow stress to the strain rate during plastic deformation. Given certain deformation conditions, its calculation formula is as follows [18]:

(3) $$m={\left(\partial \ln\sigma /\partial ln\dot{\varepsilon }\right)}_{T,\varepsilon }$$

where *m* is the strain rate sensitivity, *σ* represents the true stress, and $\dot{\varepsilon }$ represents the true strain rate. In this study, the *m* values were calculated using the flow stresses corresponding to the true strain of 0.1, and the values are presented in Table 3. Based on Equation (1), the logarithmic values of the corresponding true stresses at different strain rates were determined and are shown in Table 4. Ultimately, by using the logarithmic values corresponding to different strain rates, the *m* values for the ES and ESS samples at different temperatures were obtained and are presented in Table 5.

### 3.2. Microstructure After Hot Deformation

**Figure 3:**
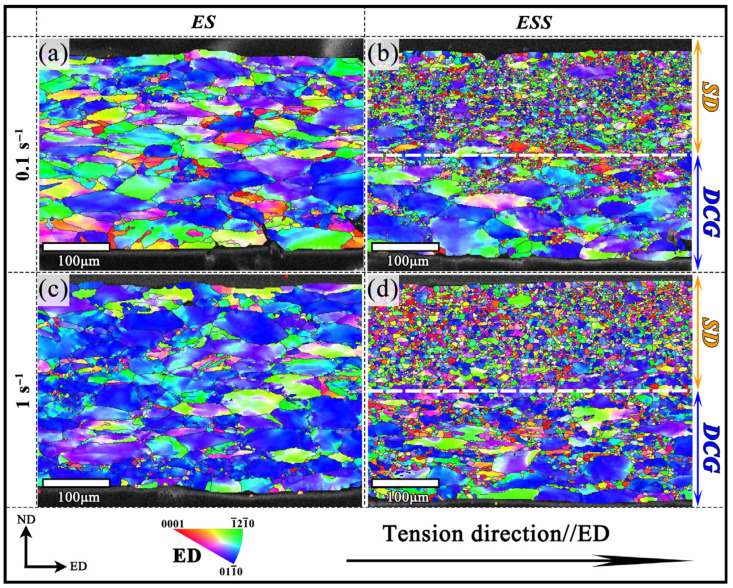
IPF plots of ES specimens and ESS samples at 400 °C: (**a**) ES Samples at 0.1 s^−1^; (**b**) ESS Samples at 0.1 s^−1^; (**c**) ES Samples at 1 s^−1^; (**d**) ESS Samples at 1 s^−1^.

Based on the concept of energy conservation, cost reduction, and preventing surface oxidation, the deformation conditions with relatively low temperature and high strain rate are preferred. Besides, normally a ductility of ~0.6 or more for alloys is expected to be sufficient for most of the forming applications where the geometry of the component is not too complex [19]. Accordingly, in this study, the ES and ESS samples deformed at 400 °C with strain rates of 0.1 s^−1^ and 1 s^−1^ were chosen for microstructure characterization to analyze deformation mechanisms. The ND-ED section inverse pole figure (IPF) maps of the ES and ESS samples deformed at 400 °C under different strain rates, along the ED projection, are shown in Figure 3. For the ES samples (Figure 3a,c), it can be observed that the coarse deformed grains were elongated along the tensile direction with a few dynamic recrystallized (DRX) fine equiaxed grains distributed around them. The average DRX grain sizes under deformation conditions of 400 °C/0.1 s^−1^ and 400 °C/1 s^−1^ were measured to be 13.56 and 12.81 μm, respectively. The microstructures of the ES specimens showed a negligible difference between the two deformation conditions.

The IPF maps for the ESS sample at strain rates of 0.1 s^−1^ and 1 s^−1^, as shown in Figure 3b,d, exhibit a gradient grain structure due to the SMAT. The microstructure can be divided into two layers based on the depth from the surface treatment: the severely deformed (SD) layer with a depth range of approximately 0~300 μm, and the deformed coarse-grained (DCG) layer with a depth range of approximately 300~600 μm [16]. The SD layer was mainly composed of a large number of fine grains, which may be due to the occurrence of recrystallization during tension [20]. In contrast, the DCG layer largely retained the CG structure similar to that of the ES samples.

**Figure 4:**
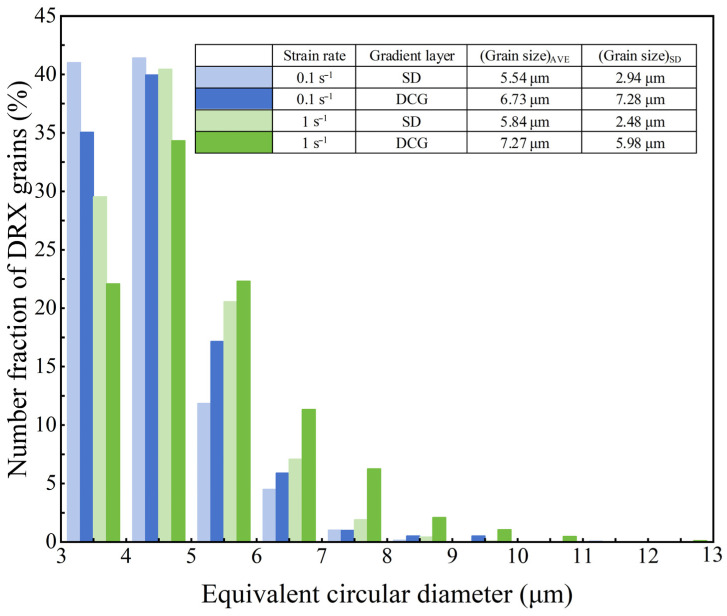
DRX Grain Size Distribution in Different Layers of ESS Samples under Various Strain Rates.

Due to the significant difference in grain structure between the different layers of the ESS sample, the grain sizes were analyzed separately for the different layers at different strain rates. As shown in Figure 4, the DRX grain size distribution of the two layers at strain rates of 0.1 s^−1^ and 1 s^−1^ demonstrates that increasing the strain rate resulted in grain coarsening. This can be attributed to the enhanced deformation heating at higher strain rates, which provides additional thermal energy for DRX grain growth [21].

## 4. Discussion

### 4.1. DRX Behavior

**Figure 5:**
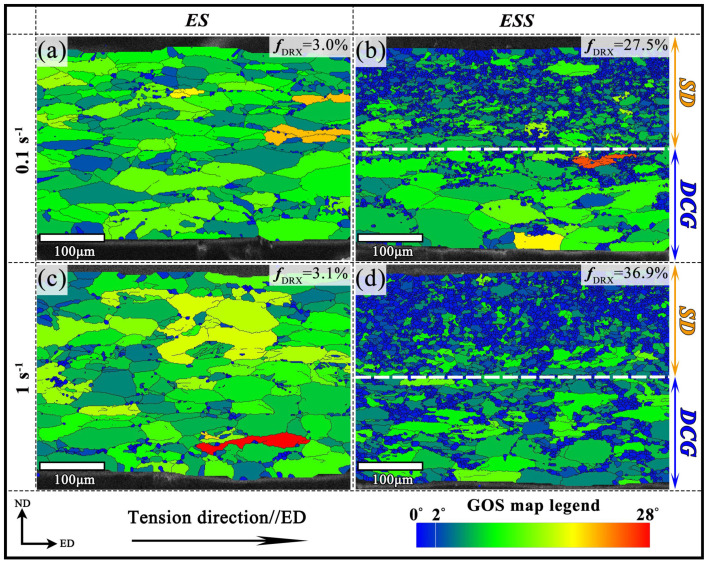
GOS maps of ES and ESS Samples at 400 °C: (**a**) ES Samples at 0.1 s^−1^; (**b**) ESS Samples at 0.1 s^−1^; (**c**) ES Samples at 1 s^−1^; (**d**) ESS Samples at 1 s^−1^.

In general, dynamic recrystallization (DRX) usually occurs during the high-temperature deformation of magnesium alloys [22,23]. In this study, the grain orientation spread (GOS) was used to identify and characterize the recrystallized grains and the deformed grains. The GOS in each grain can be expressed as the average misorientation angle between all points within the grain, as shown in Equation (4):

(4) $$GOS(i)=\sum_{j}\omega_{ij}/J(i)$$

where *J*(*i*) is the number of scanning points within the grain *i*, and *ω_ij_* represents the misorientation angle between the orientation of each scanning point within grain *i*. Generally, there is a high GOS value in a significant plastic deformation region. The GOS value less than 2° was selected to distinguish DRX grains from the deformed microstructure in this paper [24,25]. Figure 5 shows the GOS maps of the ES and ESS samples. The fine equiaxed grains can be identified as DRX grains according to the GOS values. It can be observed that the DRX degree of ES samples deformed at 400 °C at 0.1 s^−1^ and 1 s^−1^ were both extremely low (Figure 5a,c), with the area fraction of DRX grains of 3% and 3.1%, respectively. The DRX behavior of ES samples was hardly affected by increasing the strain rate from 0.1 to 1 s^−1^.

**Figure 6:**
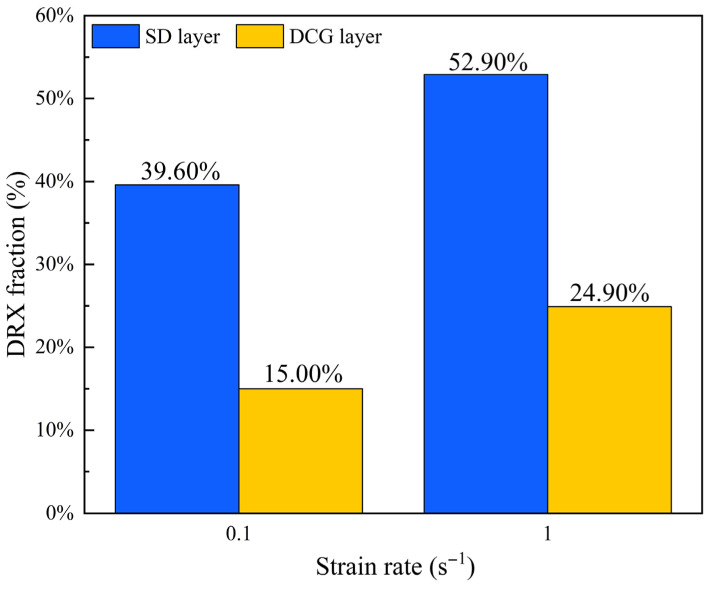
Recrystallization ratios of different layers at different strain rates in the ESS samples.

Figure 5b,d shows the GOS maps for the ESS sample deformed at 400 °C at 0.1 s^−1^ and 1 s^−1^, respectively. It is evident that there were a large number of DRX grains in the SD layer, which may be due to the presence of a high dislocation density in the SD layer by the SMAT process. The dislocation energy storage accelerated the DRX process during subsequent tensile deformation [26,27]. The DRX fractions of the SD layer and the DCG layer are shown in Figure 6. The DRX fractions of the SD layer at 0.1 s^−1^ and 1 s^−1^ were both markedly higher than those of the DCG layer, indicating a substantial DRX driving force in the SD layer. On the other hand, when the strain rate increased from 0.1 s^−1^ to 1 s^−1^, the DRX fractions of both the SD and DCG layers of the ESS samples increased. This can be attributed to the dislocation multiplication inside the material caused by the high strain rate deformation, which promoted the occurrence of DRX [28]. Furthermore, the DRX grain growth represents another contributing factor for DRX fractions increasing at 1 s^−1^ (Figure 4). It is worth noting that there were higher DRX fractions in the DCG layer compared with the ES samples. This may be related to the mechanical incompatibility between the different layers of the GS sample during deformation. It has been well known that the stress/strain partitioning between the hard/soft domains in heterogeneous structure materials could lead to improved strengthening and strain hardening [29]. However, it still needs further study whether and how the deformation incompatibility between the different layers in the GS sample affects the DRX behavior when deformed at high temperatures.

### 4.2. Residual Strain Analysis

**Figure 7:**
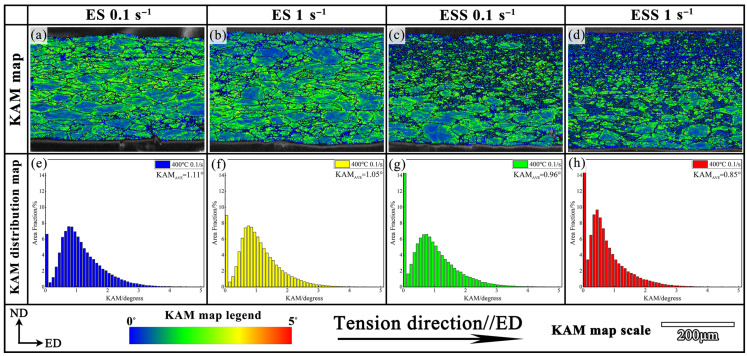
KAM maps and KAM distributions for ES and ESS samples under various strain rates: (**a**–**d**) KAM maps of ES-0.1 s^−1^, ES-1 s^−1^, ESS-0.1 s^−1^, and ESS-1 s^−1^; (**e**–**h**) KAM distributions of ES-0.1 s^−1^, ES-1 s^−1^, ESS-0.1 s^−1^, and ESS-1 s^−1^.

In order to demonstrate the residual strain and dislocation density of the experimental alloy during tensile deformation, the Kernel Average Misorientation (KAM) and grain boundary (GB) distribution were investigated [30,31]. The KAM reflects the strain distribution by calculating the average misorientation between each point and its surrounding points [32], as shown in Equation (5) [33]:

(5) $$KAM(\mathrm{point}j)=\sum_{i=1}^{N}\omega \;(g_{i},g_{j})/N$$

where *N* is the total number of adjacent EBSD points that satisfy the threshold misorientation value (usually 5°), and *ω* (*g_i_*, *g_j_*) is the misorientation angle between the point *j* and adjacent point *i*. Figure 7 shows the KAM maps and KAM distributions for the ES and ESS samples at 400 °C under different strain rates, respectively. The yellow regions around the boundaries of large-sized grains represented high-strain concentration associated with high dislocation density, while the blue regions in the DRX grains indicated the low strain distribution and low dislocation density. According to the KAM distribution (Figure 7e–h), the ESS sample had the lowest average KAM value (KAM_Avg_, 0.85°), suggesting that DRX grain nucleation and growth alleviated strain concentration caused by dislocation accumulation at grain boundaries. In addition, as the strain rate increased from 0.1 s^−1^ to 1 s^−1^, the KAM_Avg_ values of both ES and ESS samples slightly decreased, indicating that more dislocations were consumed by DRX at higher strain rates.

**Figure 8:**
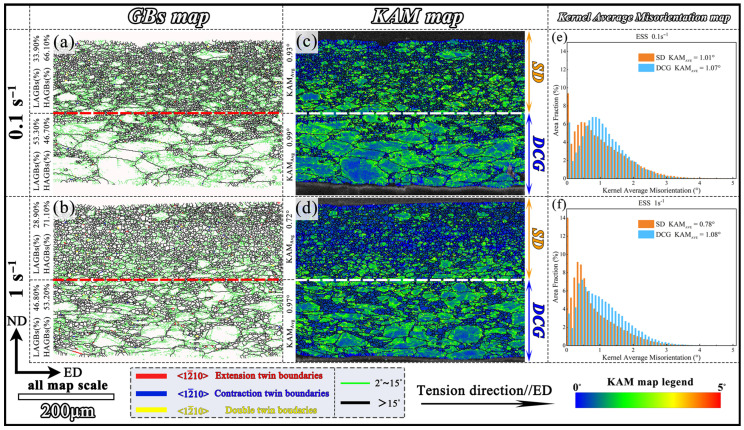
(**a**,**b**) Grain boundary maps, (**c**,**d**) KAM maps, and (**e**,**f**) KAM distributions of ESS samples at 400 °C: (**a**,**c**,**e**) 0.1 s^−1^; (**b**,**d**,**f**) 1 s^−1^.

Further analysis was conducted on the grain boundaries and KAM distribution maps of different layers of the ESS samples, as shown in Figure 8. The low-angle grain boundaries (LAGBs, 2°~15°) were mainly distributed within the deformed grains, especially in the DCG layer, consistent with the distribution of high KAM values. During the hot deformation, dislocations accumulated inside the grains and further transformed into LAGBs by dislocation rearrangement. Due to the large fraction of DRX grains, the SD layers had more high-angle grain boundaries (HAGBs, >15°) than the DCG layers (66.1% vs. 46.7% at 0.1 s^−1^ and 71.1% vs. 53.2% at 1 s^−1^).

### 4.3. Deformation Mechanisms

**Figure 9:**
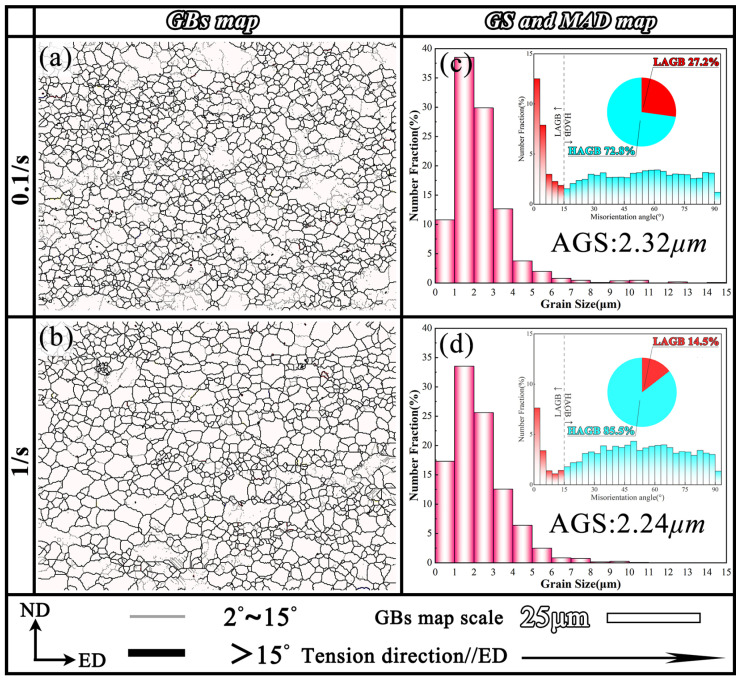
The microstructure in the SD layer at strain rates of 0.1 s^−1^ and 1 s^−1^: (**a**,**b**) GBs maps; (**c**,**d**) grain size, HAGB, and LAGB distributions.

Figure 9 presents the fine-grain microstructure in the SD layer at strain rates of 0.1 s^−1^ and 1 s^−1^, which primarily consists of small, uniformly distributed DRX grains. The corresponding grain size, HAGB, and LAGB distributions are displayed in Figure 9c,d. The grain size is predominantly concentrated around 2 μm, with 49.28% of grains below 2 μm at 0.1 s^−1^ and 50.83% at 1 s^−1^. Furthermore, the HAGB fractions were measured to be 72.8% at 0.1 s^−1^ and 85.5% at 1 s^−1^, which was expected to facilitate grain boundary sliding (GBS) during plastic deformation, as a high proportion of HAGBs would reduce slip energy barriers and increase strain rate sensitivity [34].

GBS is generally considered as one of the important deformation mechanisms at high temperatures, effectively improving the deformability of magnesium alloys [35]. Lots of studies [36,37,38] reporting superplastic deformation behaviors by GBS mechanism in Mg alloys adopted low strain rates (≤10^−3^ s^−1^). This may be related to the inadequate rate of grain boundary diffusion at high strain rate (e.g., 0.1 s^−1^) [11]. However, superplastic behaviors were achieved in WE43 Mg alloy when deformed at 350–450 °C and at a high strain rate of 0.1 s^−1^. That was attributed to the very fine-grained size of ~340 nm in their work. Other studies [39,40,41] also reported superplasticity in Mg alloys deformed at a high strain rate of 0.1 s^−1^ when grain sizes were refined to ~1.5 μm. Thereby, the considerable amount of fine grains smaller than 2 μm in the SD layer of the present GS alloy could cause GBS when deformed at strain rates ≥ 10^−2^ s^−1^. Moreover, the high fraction of low residual-strain equiaxed fine grains in the SD layers, revealed by the KAM maps (Figure 8), implied that GBS accompanied by DRX played important roles during the high-strain-rate tensile tests [11].

In order to reveal the deformation mechanisms in the coarse grains of the DCG layer of ESS sample and in the ES sample, IGMA analysis was performed based on the EBSD results, which was achieved by matching the theoretical slip system axis (Taylor axis) with the LAGB misorientation axis inside the grains [42]. By simply aligning the experimentally measured IGMA with the Taylor axis, the primary mechanism of deformation in the deformed grains can be identified [31,43,44]. The Taylor axis (*T*_s_) is defined as [45]:

(6) $$T_{s}=n_{s}\times d_{s}$$

where *n*_s_ and *d*_s_ are the slip plane normal and slip direction for a given slip system. Based on Equation (4), the Taylor axis and deformation mechanism corresponding to the slip system in magnesium alloys are shown in Table 6. Based on the Taylor axis of the given slip system and the experimentally measured IGMA, the slip mode in the dominant deformed single crystal can be determined. For example, IGMA distribution concentrated along the <0001> direction is commonly considered the result of prismatic <a> slip, while IGMA distribution inclined towards the <uvt0> direction is associated with basal <a> slip or Pyramidal <c + a> II slip [46].

**Figure 10:**
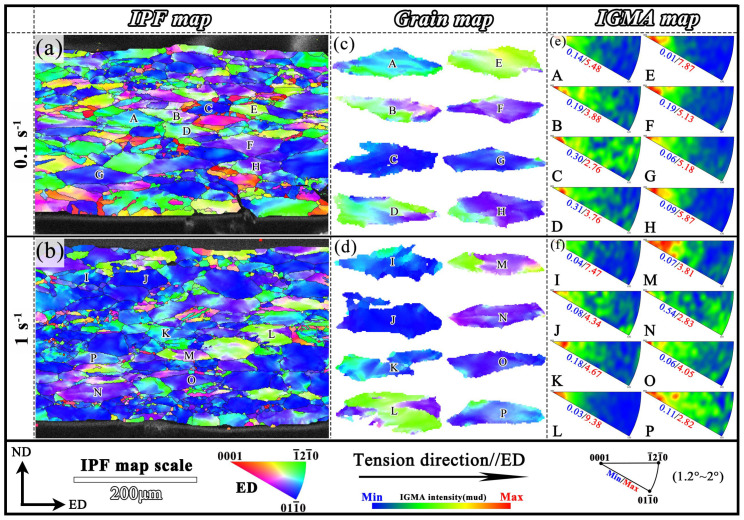
IGMA analysis of ES Samples: (**a**,**b**) IPF maps; (**c**,**d**) grain maps; (**e**,**f**) IGMA maps.

**Figure 11:**
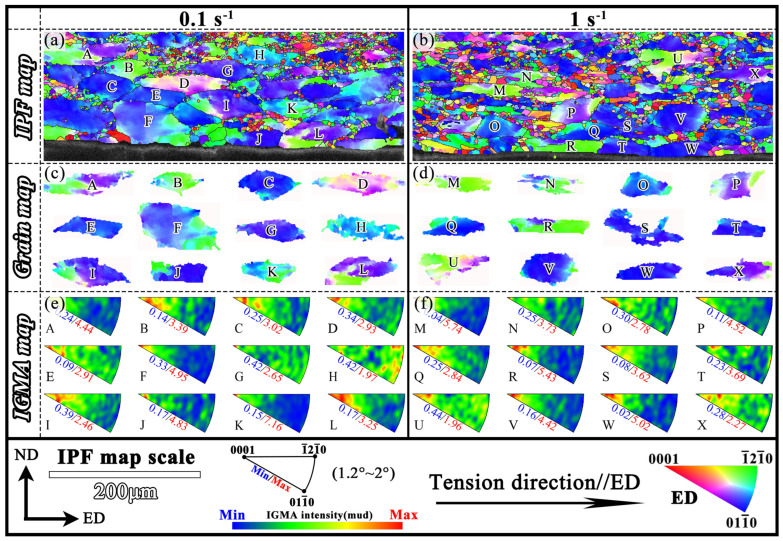
IGMA analysis of ESS Samples: (**a**,**b**) IPF maps; (**c**,**d**) grain maps; (**e**,**f**) IGMA maps.

The IGMA maps based on EBSD of ES samples and the DCG layer in ESS samples are shown in Figure 10 and Figure 11. Previous studies have reported that the misorientation angle less than 1.2° may lead to apparent misorientation due to the limited angular resolution of EBSD. While the misorientation angle above 2° showed a negligible effect on the IGMA qualitative distribution characteristics [46,48]. Accordingly, all the IGMA distributions shown in this study were considered for the misorientation axes ranging from 1.2° to 2° [42,46,49]. For the ES samples, the 8 grains with large deformation at 0.1 s^−1^ and 1 s^−1^ were selected as the research objects (Figure 10), respectively. It can be seen that the IGMA distributions for all the grains were concentrated around the <0001> axis. Thus it can be inferred that the grain deformation mechanism in the ES samples was primarily governed by prismatic <a> slip. The singular prismatic slip mechanism in coarse deformed grains could lead to inadequate deformation capability observed in the ES samples. Generally, the activation of prismatic slip system is highly sensitive to crystallographic orientation [48]. Additionally, due to the higher critical shear stress (CRSS) required for the activation of prismatic slip and the poor geometric compatibility between its slip direction and slip plane, the CG region is more prone to unfavorable orientation during deformation, which would impede slip transmission, leading to local stress concentration and promoting the initiation of microcracks [50].

For the ESS sample, 12 deformed grains (GOS > 2°) were randomly selected from the DCG layer of the two samples at 0.1 s^−1^ and 1 s^−1^ for IGMA analysis, as shown in Figure 11. The distribution of maximum intensity (MI) can indicate the slip behavior in deformed grains. When MI < 2 mud, the grains have uniform IGMA and no dominant slip system, related to small plastic deformation. When MI > 2 mud, it suggests that there is preferential IGMA in the grain and that specific slip systems predominate inside the grain, which indicates large plastic deformation inside the grain [51]. At 0.1 s^−1^, the H grain shows the MI below 2 mud (Figure 11a), indicating that no preferential slip mechanism was present and the deformation was dominated by multiple slip systems. The IGMA distributions for the other grains were mainly concentrated around the <0001> axis, indicating that the deformed grains in the DCG layer of the ESS sample at 0.1 s^−1^ primarily deformed by prismatic <a> slip. Similarly, Figure 11b shows that, except for the IGMA distribution of grain U showing MI below 2 mud, the IGMA distribution of other grains was mainly concentrated near the <0001> axis, indicating prismatic <a> slip predominated. Thus, the deformation grains of DCG layers at 0.1 s^−1^ and 1 s^−1^ were mainly dominated by prismatic slip.

Based on the above IGMA analysis, the Taylor axes of most deformed coarse grains were along the <0001> direction, indicating the main slip mode was prismatic <a> slip. During the high-temperature tension, a single deformation mechanism cannot effectively coordinate the plastic deformation, which could cause local strain concentration and limited deformability. As identified by Kardani et al. [52] via molecular dynamics simulations, the limitation of dislocation movement in the stacking fault is the fundamental micromechanism responsible for local strain concentration, which in turn enables sources of dislocation. Local strain concentration is further exacerbated by the concentrated porosities, which promote dislocation accumulation and the consequent formation of local shear bands, ultimately leading to material embrittlement.

Xiao et al. [53] reported a high elongation of 77% when a coarse-grained Mg-9.0Gd-4.0Y-0.6Zr (wt%) alloy (with average grain size of 40 μm) was tensioned at 350 °C and at a strain rate of 4.6 × 10^−4^ s^−1^, which was attributed to basal and non-basal dislocation glides. They also reported an elongation of 180% at 400 °C and at the same strain rate, and during the deformation, dislocation gliding and twinning, dynamic recrystallization played important roles. Li et al. [54] reported a superplastic behavior of 300% elongation when a coarse-grained Mg-9.0Gd-3.0Y-0.5Zr (wt%) alloy (with average grain size of 208 μm) was tensioned at 450 °C and at a strain rate of 5 × 10^−4^ s^−1^. The superplastic deformation mechanism was pointed out to be predominated by glide-controlled dislocation creep assisted by grain boundary sliding, with a strain rate sensitivity exponent *m* of about 0.32. Dynamic recrystallization during tensile testing also contributed to the superplasticity. However, neither the deformation parameters nor the deformation mechanisms in the above reports were applicable to the present ES samples. These were in line with the low *m* value and the extremely low DRX ratios of the ES samples (Table 5 and Figure 5). Wu et al. [10] reported that the *m* value of the WEZ612 alloy (average grain size: 5.22 ± 0.6 μm) is in the range of 0.16–0.35, and the *m* value is 0.16 at a low temperature of 623 K with high strain rates, which is characteristic of dislocation creep. In comparison, the *m* value (0.244) of the ESS sample under the same deformation conditions in this article is higher than that.

The tensile behaviors of the ESS samples were the overall performance of the SD layer and the DCG layer. Thereby, the *m* values of the ESS samples (Table 5) may be the integrated reflection of the deformation mechanisms of the two layers. When GBS mechanism made substantial contributions to the superplastic deformation, the *m* value could reach 0.5 or above [11,36,37,55]. However, when strain rate was increased above 10^−3^ s^−1^, dislocation motion process could operate associated with the high deformation rate, resulting in decreased *m* value (e.g., ~0.14) [37]. Therefore, by comparing the *m* values and the performances of the ESS and ES samples, it could be disclosed that the GBS associated with the DRX in the SD layer contributed predominantly to the deformation of the ESS samples, at the relatively low temperature (≤400 °C) and at the high strain rates (≥10^−2^ s^−1^). Furthermore, the interactions between the SD and DCG layers and the related effects in the GS sample during high-temperature deformation need to be studied in the future.

## 5. Conclusions

In this study, high-temperature tensile tests were conducted on the uniform coarse-grained and gradient-structured Mg-Gd-Y-Zr alloys, respectively. The deformation behavior, DRX characterization, and dislocation slip mechanisms were investigated by flow curves analysis and EBSD technique. The following conclusions can be drawn:

1. The ES samples with uniform CG structure exhibited high flow stress and low elongation, while the ESS samples with gradient structure exhibited lower flow stress and higher elongation. Moreover, the strain rate sensitivity index (*m*) values of the ESS samples were higher than those of the ES samples.
2. The ES samples exhibited significantly lower DRX ratios (3% and 3.1%) compared to the ESS samples (SD layer: 39.6% and 52.9%; DCG layer: 15.0% and 24.9%) under deformation conditions of 400 °C/0.1 s^−1^ and 400 °C/1.0 s^−1^, respectively. When the strain rate increased from 0.1 s^−1^ to 1.0 s^−1^, the enhanced deformation heating promoted DRX grain growth in the ESS samples, resulting in improved DRX.
3. The deformed grains in the ES samples contained high residual stresses, and the deformation relied solely on the prismatic <a> slips in the coarse grains. The lack of softening and coordinating effect during the deformation led to the low plasticity. In contrast, the high dislocation density in the SD layer of the ESS samples promoted profuse DRX during deformation. The GBS mechanism facilitated by DRX significantly enhanced the overall deformability of these samples.

## Figures and Tables

**Table 1:** Chemical compositions of the Mg-Gd-Y-Zr alloy.

Alloy	Compositions (wt%)			
	Gd	Y	Zr	Mg
Mg-8.5Gd-2.5Y-0.5Zr	8.34	2.32	0.35	Bal.

**Table 2:** Sample designation and the corresponding procedures.

Sample Designation	Procedures
ES	Extrusion + Solution treatment
ESS	Extrusion + Solution treatment + SMAT

**Table 3:** The true stress values at 0.1 true strain for the ES and ESS samples at different temperatures.

Samples	Temperature (°C)	True Stress at 0.1 True Strain			
		0.01 s^−1^	0.1 s^−1^	0.5 s^−1^	1 s^−1^
ES	350	228.916	143.977	106.294	58.663
	400	253.492	171.462	169.909	113.598
ESS	350	256.450	210.268	203.206	160.078
	400	277.853	221.873	244.451	178.860

**Table 4:** The logarithmic values at 0.1 true strain for the ES and ESS samples at different temperatures.

Samples	Temperature (°C)	Logarithm of the True Stress at 0.1 True Strain			
		0.01 s^−1^	0.1 s^−1^	0.5 s^−1^	1 s^−1^
ES	350	5.433	4.970	4.666	4.072
	400	5.535	5.144	5.135	4.733
ESS	350	5.547	5.348	5.314	5.076
	400	5.627	5.402	5.499	5.187

**Table 5:** The values of strain rate sensitivity index *m* for ES and ESS samples.

Samples	Temperature (°C)	*m*
ES	350	0.037
	400	0.096
ESS	350	0.173
	400	0.244

**Table 6:** List of Taylor axes corresponding to the slip system observed in magnesium alloys [47].

Slip Mode	Slip Type	Slip Systems	Taylor Axes	Variants
$\{0001\}\;<11\bar{2}0>$	Basal <a>	3	$<1\bar{1}00>$	3
$\{01\bar{1}$ $0\}\;<1\bar{2}10>$	Prismatic <a>	3	<0001>	1
$\{10\bar{1}$ $1\}\;<1\bar{2}10>$	Pyramidal <a>	6	$<10\bar{12}>$	6
$\{10\bar{1}$ $1\}\;<11\bar{23}>$	Pyramidal I <c + a>	12	$<\bar{25}$$,41,\bar{16},9>$ *	6
$\{11\bar{2}$ $2\}\;<11\bar{23}>$	Pyramidal II <c + a>	6	$<\bar{1}100>$	3

* denotes a Taylor axis valid only for an ideal c/a ratio (the other values do not depend on this ratio).

## Data Availability

The original contributions presented in this study are included in the article. Further inquiries can be directed to the corresponding authors.
